# How do topics and emotions develop in elementary school children? A text mining perspective based on free-writing text over 6 years

**DOI:** 10.3389/fpsyg.2023.1109126

**Published:** 2023-03-02

**Authors:** Mengjun Liu, Xinyu Jiang, Bingbing Zhang, Ting Song, Gang Yu, Guofang Liu, Nan Jiang, Di Wu, Zhi Zhou

**Affiliations:** ^1^School of Education, Hubei University, Wuhan, China; ^2^School of Foreign Languages, Hubei University, Wuhan, China; ^3^No. 1 Middle School of Xiaochi Town, Huanggang, China; ^4^China Unicom Hubei Branch, Wuhan, China

**Keywords:** children development, text mining, topic modeling, sentiment analysis, ecological systems theory

## Abstract

Free-text data with long duration and continuity have great potential for studying environmental concerns and emotional expressions in child development. Based on ecosystem theory, using topic modeling and sentiment analysis in text mining to mine 4556 free-text writing data from first to sixth grade in elementary school, this study aims to reveal concerned topics’ evolutionary trends and sentiment expression differences in topics during elementary school children’s development. The results show the following: (1) Children prefer to focus on the topics of school and family in elementary school; (2) With the growth of grades, the proportion of family topics continues to decline, while that of social culture topics keeps rising; (3) When describing school, family, social culture, and interest, children mostly express negative emotions, and when describing peers and ability they mostly express positive emotions; (4) As the grade increases, the emotional expression on social culture topics become negative, while that on ability and interest topics become positive, and there are more differences in emotion expression between topics in junior and senior elementary grades. Discussion and conclusion are discussed at the end.

## 1. Introduction

The ecological systems theory suggests that the ecological environment in which children develop is composed of several interlocking systems (Bronfenbrenner, 1977). The development of an individual is not only reflected in the influence of a single micro system on its development but also the role of various hierarchical systems directly or indirectly related to the individual. In this way, the individual adapts and develops from family to the external environment and then to the larger social environment (Miyamoto et al., 2015; Primi et al., 2016). Studies of the different systems and interactions between systems that affect children’s development are often performed using closed or graded questionnaires and self-reports. In addition to these data sources, ubiquitous textual data bring new perspectives and great potential for researchers to explore how children adapt and develop. Because studies have shown that text data can truly reflect an individual’s conceptions, emotional attitudes, and feelings (Witten et al., 2011). Text mining can extract meaningful knowledge or patterns from unstructured text documents, and has been widely used and researched in discovering users’ behavior, cognition and sentiment. Therefore, this study aims to mine the changing characteristics of environmental influences and emotional expression in children’s development from textual data.

By combining machine learning and information retrieval techniques, text mining can discover and extract hidden and previously unknown knowledge from large amounts of text data, and ultimately form valuable knowledge (Feldman and Dagan, 1995). Text mining mainly includes two parts: topic modeling and sentiment analysis. Topic modeling utilizes machine learning and natural language processing algorithms to locate and count keywords, and identify hidden or unobserved targets and topics through keyword proportions and associated structures. These abstract topics are important factors to explain or influence individual willingness and behavior changes. Common examples are e-commerce reviews (Liu et al., 2017), social media texts (Sun et al., 2020), e-Learning discussions, and feedback (Nilashi et al., 2022). Sentiment analysis can identify and extract subjective information in documents and is often used to help discover users’ opinions, attitudes, and emotional tendencies. Two emotional dimensions namely positive and negative are commonly used in sentiment analysis and are often related to user motivation and satisfaction (Hew et al., 2020; Sun et al., 2020). Detecting users’ sentiment expressions under different topics can help decision-makers identify and intervene in negative emotions and avoid the negative impact of topics on users (Liu S. et al., 2019). In recent years, the combined use of topic modeling and sentiment analysis to study hidden topics and sentiment tendencies in short texts has become a popular trend in text-mining research. In particular, adding a temporal dimension can be used to investigate the topic and sentiment development changes. For example, based on course forum text data, Liu Z. et al. (2019) and Peng et al. (2020) dynamically tracked students’ topic evolution and topic sentiment expressions, to understand their learning needs. Nonetheless, text-mining researches on topics and sentiments in children’s development are still relatively insufficient in terms of its macro-time scales. This is due to the difficulty of obtaining free text data over long periods and continuity while avoiding research interference with children’s expressions.

In this study, we collected long-term, continuous free-text data in collaboration with a senior elementary school language teacher over up to 6 years. Data came from primary school children with an unlimited theme “Today I want to say…,” to record children’s observations and reflections on their daily life. As there are no restrictions on content, format, and skills, the data have the characteristics of no restriction and free play. We believe that the 6-year data can help enable longitudinal studies investigating changes in primary school children’s focus on topics and emotions. Because, generally speaking, textual data on children’s development are primarily generated during learning and social activities, including learning discussions, curriculum feedback, subject assignments, and social media. Discussions, feedback, and assignments about learning are limited by subject context (Xing et al., 2020), and cannot fully reflect the daily life of children. With the widespread use of the social Internet, some scholars believe that texts of thoughts, opinions, and feelings freely expressed on the Internet by children and adolescents, can be used to study changes in their development process. For example, Hipson (2019) used sentiment analysis on a sample of 8,688 poems published online by children and adolescents in grades 4 through 12 to identify some changing patterns of children’s emotional expression. However, the online texts in this type of research are only cross-sectional data of multiple groups of children and adolescents of different ages at a certain point in time. Strictly speaking, it is not data on topic and sentiment expression over time, and it is not possible to fully analyze the trend of children’s concerned topic and sentiment expression. In comparison, the free-writing text data of the same group of primary school students collected through 6 years continuously in this study can better reflect the development trends of children’s daily life concerns and emotional expression.

In summary, based on a collected writing text dataset with a long period and free-play characteristics, this paper uses topic modeling and sentiment analysis to study the children’s changes in concern topics and differences in sentiment expressions from grade 1 to grade 6. The study attempts to contribute to the growing knowledge base of child development research based on ecological systems theory and child emotional development research. At the same time, it can help parents and teachers to effectively guide and meet the emotional concerns of elementary school children for counseling, thus better guiding children’s growth promptly and helping to promote their psychological health development.

## 2. Literature review

### 2.1. Text mining in learning analytics

The growth of online learning and information technology has provided researchers with a wealth of textual data. Common sources of text contain course reviews, learning discussions, social interactions, and instructional documents. Topic modeling can mine the semantic information in the text, revealing the learner’s cognitive engagement process and the influencing factors that affect meaningful learning. For example, Nilashi et al. (2022) used topic modeling for course reviews to discover course-related dimensions and identify factors that affect learner satisfaction. Liu S. et al. (2022) used the temporal cognitive topic model to investigate the discussion topics of different achievement groups in the cognitive engagement model, which helped teachers to provide guidance and timely intervention in the MOOC. In addition to identifying hidden and dominant topics in texts, tracking long-term textual data using topic modeling can also help uncover development patterns and trends. For example, Xing et al. (2020) used topic modeling for students’ subject homework texts and found the semantic patterns of students’ scientific argumentation. Southavilay et al. (2013) used topic modeling to track topic changes during students’ collaborative writing to help teachers effectively monitor groups and find problems early. In recent years, several researchers have used sentiment analysis to identify learners’ learning experiences and discover the relationship between students’ sentiments and learning engagement. Li et al. (2022) and Xing et al. (2019) conducted sentiment analysis for course reviews to predict learner satisfaction and completion rates for MOOC. Liu Z. et al. (2022) used sentiment analysis for MOOC forum discussions and found that positive and confused emotions are correlated with higher-level cognitive engagement. Peng and Xu (2020) used the Behavior-Emotion Topic Model to identify learners’ focused topics and topics’ emotional tendencies in reviews, discovering the behavior patterns of completers and non-completers. Text mining has gained increasing attention in researching cognition and emotion, especially in longitudinal investigations of learners’ cognitive development patterns and emotional development patterns. Therefore, this study unites topic modeling and sentiment analysis to analyze free-writing data of primary school children and to investigate children’s concern topics and emotional tendencies of topics in elementary school grades 1–6.

### 2.2. Ecological systems theory

As one of the most widely used theoretical frameworks to study children’s development, ecological systems theory suggests that the ecological environment of individual development is a nested structure from micro to macro (Bronfenbrenner, 1977). Specifically, Bronfenbrenner’s ecological model includes microsystem, mesosystem, exosystem, and macrosystem. Microsystem refers to the surrounding environment that directly interacts closely with the individual. Family, school, and peers are the most common microsystems. Mesosystem refers to the interaction of various elements within the microsystem. Exosystem refers to the external environment that does not directly or closely contact the individual but affects the individual. Macrosystem refers to broader social systems and cultural concepts. Ecological systems theory is often used to identify individual systems that affect children’s development and the interactions between systems, to explain a range of phenomena. For example, Paat (2013) examined the impact of families, peers, schools, neighborhoods, immigrant culture, and values on the adolescent lives of mainstream immigrant children in the United States. Serpell and Mashburn (2012) examined the importance of family–school connections (the frequency of parent-teacher contacts and the quality of parent-teacher relationships) to children’s early social development. Bluteau et al. (2017) investigated the interplay of individual, curriculum and institution, community, national context, and culture in students’ cross-disciplinary learning development. Children’s development is not only affected by the dynamically changing ecological environment but also by their characteristics, traits, physiology, psychology, and interest are important attributes that shape the outcome of children’s development (Stokols, 1996). Therefore, this study uses topic modeling based on ecological systems theory to uncover the environmental and individual factors involved in the text data, helping to understand how children’s concerns extend from the home environment to the larger social environment during the elementary school years.

### 2.3. Developmental change in children’s emotion

Studies of emotional development in childhood and adolescence are often understood in terms of changes in affective valence (i.e., positive or negative) and magnitude. Children and adolescents are mostly positive in their daily states (Larson and Lampman-Petraitis, 1989). However, in early to middle adolescence, the average emotional state becomes more negative over time, with more negative emotions and fewer positive emotions (Larson et al., 2002). A subsequent longitudinal study showed that decreases in positive affect persisted into late adolescence, but the negative affect is relatively stable. The findings also indicated that the decrease in average emotions in late adolescence is attributable to a continued deterioration of positive mood rather than an increase in negativity (Weinstein et al., 2007). The changing living environment is an important factor in explaining changes in the emotional development of children and adolescents. After entering primary school, children interacted less with the family environment and had more conflicts with their parents. Teachers play an important role in the early years of elementary school, and the quality of the teacher-student relationship could affect children’s emotional experience and academic achievement at school (Spilt et al., 2012; Hajovsky et al., 2017). As children and adolescents grow, they would become more independent and less dependent on teachers, and increasingly they value their peers’ support and their motivations and abilities more (Maldonado-Carreño and Votruba-Drzal, 2011). Peer-friendship quality links to higher positive emotions and junior anxiety (Schneiders et al., 2007). Meanwhile, children and adolescents have increased perceptions of negative events (Larson and Ham, 1993), including family conflicts, peer relationships, and concerns about social evaluations which all contribute to increased negativity and anxiety (Dong et al., 1994). Although a growing body of research has focused on discovering and explaining emotional development changes in children and adolescents in different stages of context, longitudinal studies are still rare. Therefore, this study aims to use sentiment analysis to reveal the development changes of children’s focus on topics and emotional tendencies, and to enrich the research on children’s emotional development.

## 3. Materials and methods

### 3.1. Privacy guarantee

The purpose and content of the study had been fully explained to school teachers, students, and parents, who were free to agree or refuse without prejudice. Data were desensitized using anonymous IDs during processing to ensure the security and protection of personal privacy. All data were properly obtained with the informed consent of those involved.

### 3.2. Data collection and pre-processing

We collected free-writing text data from 18 students, including eight boys and 10 girls who studied between the years 2011 and 2017 from Grade 1 to Grade 6 of Primary School C, City W, Hubei Province, People’s Republic of China. The 18 students were taught Chinese lessons by the same teacher during the 6 years of primary school. The teacher encouraged the students to record their observations and thoughts about their daily life in the form of a diary called “What I want to say today.” Through digital processing, data cleaning, and sorting, a total of 4,576 valid free-writing articles were obtained, with an average of 254 articles per student, with an average of about 163 words per article, of which 1,988 pieces were from boys and 2,588 were from girls.

### 3.3. Data analysis method

In this study, we used the LDA model to mine the topics of free-writing text data, and combined with ecological systems theory to classify and discuss the topics to analyze the topics of children’s concerns and the trends of the topics at the elementary school. The ecological systems theory emphasizes the different systems that influence children’s development. Topic analysis can reflect the role and influence of different systems in children’s development through the proportion and change trend of topics. LSTM is used to mine the sentiment tendency of text data under different topics. The Kruskal-Wallis test was used to examine sentiment expression changes as grade level increased, and sentiment expression differences on different topics within the same grade level.

The Latent Dirichlet Allocation (LDA) model (Blei et al., 2003) is a probabilistic model that can mine potential topics from a large set of documents, with good scalability. This study implemented the LDA model in Python’s Gensim package. Specifically, the document collection is preprocessed first, including word segmentation and removal of stop words, to obtain a sequence of phrases; then it assigns ID to each word, and organizes the word frequency, using “word ID: word frequency” to convert it into a sparse vector format that can be processed by Gensim; finally, the parameters related to the number of topics and the number of keywords are adjusted to determine the LDA model, mainly using the similarity in the returned results to determine the optimal number of topics, and the coherence to evaluate the interpretability of the topics. The LDA model is used to analyze the “Today I want to say” free-writing text data, thereby determining the keywords and word frequency relevant to the topic, which experts and teachers together evaluate to determine specific topics. And then according to the ecological systems theory, the obtained topics are further classified and analyzed.

The Long Short-Term Memory (LSTM; Hochreiter and Schmidhuber, 1997) based text sentiment analysis has achieved good results in capturing the information of the deep structure of the text and the dependencies among the texts, and in analyzing and processing long sequences and long-term dependent data. This study used python to utilize the sentiment analysis API^1^ openly shared by the deep learning company Paralleldots, to achieve rapid automatic processing of large batches of text. The API uses the LSTM to classify the sentiment of text into three categories: Positive, Negative, and Neutral. Single text analysis results of the data include the overall sentiment type and the proportion of the three emotion types. For example, the overall sentiment type of “feeling good after running in the morning” is classified to be positive, specifically with a proportion of 74.9% positive, 20.90% neutral, and 4.10% negative. This research only focuses on the overall sentiment type of each free-writing text for elementary school children, using “2” for positive, “1” for neutral, and “0” for negative.

## 4. Results

### 4.1. Topic modeling results

By adjusting relevant parameters and conducting several experiments and comparisons, this paper found that when the number of topics is *K* = 14 and the number of keywords is 8, the similarity of each topic is smaller and the topics are better clustered. According to the proportion and associated structure of each keyword, the study determined the naming of 14 topics, including class, daily, family, dream, like, civilization, entertainment, score, ability, news, food, story, homework, and play, as shown in Table 1. To determine the topic of each writing, the writing of topic probability of more than 0.3 was set as belonging to the topic. Overall, class (*N* = 1,579, 30.5%) and daily (*N* = 694, 13.4%) accounted for more, and homework (*N* = 85, 1.6%) and play (*N* = 58, 1.1%) accounted for less. Class topics included descriptions of course activities, teacher-student interaction, and student–student interactions, while family topics focus on descriptions of family members’ daily activities.

**Table 1:** Topics and subject words.

Topic name	Subject words	Number and proportion of posts	
Class	Teacher, Pan (Last name of class teacher), classmate, do, write, think, homework, and study	1,579	30.5%
Daily	Mom, dad, say, think, do, go home, night, and walk	694	13.4%
Family	Say, mom, grandma, play, sister, eat, think, and dad	486	9.4%
Dream	Book, think, like, hope, sky, book, happy, and paint	343	6.6%
Like	Like, cute, one animal, eyes, gift, grass, cat, and birthday	338	6.5%
Civilization	China, garbage, test, month, civilization, doing, news, and hope	321	6.2%
Entertainment	Spring, beauty, birds, classmate, campus, playground, animals, and school	312	6.0%
Score	Score, deduct, 100, speak, do, 10, 20, and weekly	267	5.2%
Ability	Competition, speak, act, run, train, piano, sports meeting, and ball	232	4.5%
News	Father, son, life, run, doctor, news, fog, and happen	172	3.3%
Food	Eat, apple, like, delicious, life, fish, eat, and stomach	160	3.1%
Story	Old man, say, grandpa, year, hair, like, love, and internet	125	2.4%
Homework	Recite, little white rabbit, traditional Chinese culture, inspection, arrangement, tortoise, preview, and the analects of Confucius	85	1.6%
Play	Day, song, little apple, spring outing, happy valley, Chongqing, childhood, and play	58	1.1%

Ecological systems theory emphasizes that individuals are nested in a series of interacting environmental systems, and the interaction of environmental systems and individual factors influences the development of individuals. According to the ecological system theory, we organized and merged the above 14 topics from the two dimensions of environmental system and individual factors into six topics of school, family, peer, social culture, ability, and interest, as shown in Table 2. By removing text data that had no attached topic and adding those that were subject to many topics, the final total sample size reached 5,014 articles. Among them, the school topic texts mainly described interactions between teachers and classmates in the school environment, and school activities involving learning, communication, and play. The family topic texts mainly involved the interactions between children and family members. The peer topic texts mainly talked about activities between children and their peers in and out of school. The social culture topic texts mainly focused on children’s understanding and feelings about social news, traditional Chinese festivals, and the experiences of their elders. The ability topic texts were mainly related to children’s expectations and the ability they have learned. The interest topic texts were more about children’s descriptions and attitudes toward things, food, and the environment.

**Table 2:** Classification of topic categories and subject words.

Perspective	Topic name	Construct	Subject words	Number and proportion of posts	
Environmental system	School	Class, score, and homework	Teacher, Pan, classmate, homework, study, class, write, mark, deduct, and recite	1847	36.8%
	Family	Daily, family	Mom, dad, home, night, grandma, play, sister, night, morning, and walk	1,128	22.5%
	Peer	Entertainment, play	Spring, beautiful, bird, Classmates, campus, animals, forest, spring outing, happy valley, and Chongqing	366	7.3%
	Social Culture	News, civilization, and story	China, garbage, civilization, news, country, father, son, child, read, and life	607	12.1%
Individual factors	Ability	Ability, dream	Book, contest, sun, acting, run, training, think, wish, and like	572	11.4%
	Interest	Like, food	Like, cute, eyes, gift, grass, cat, apple, fish, meal, and stomach	494	9.9%

**Figure 1:**
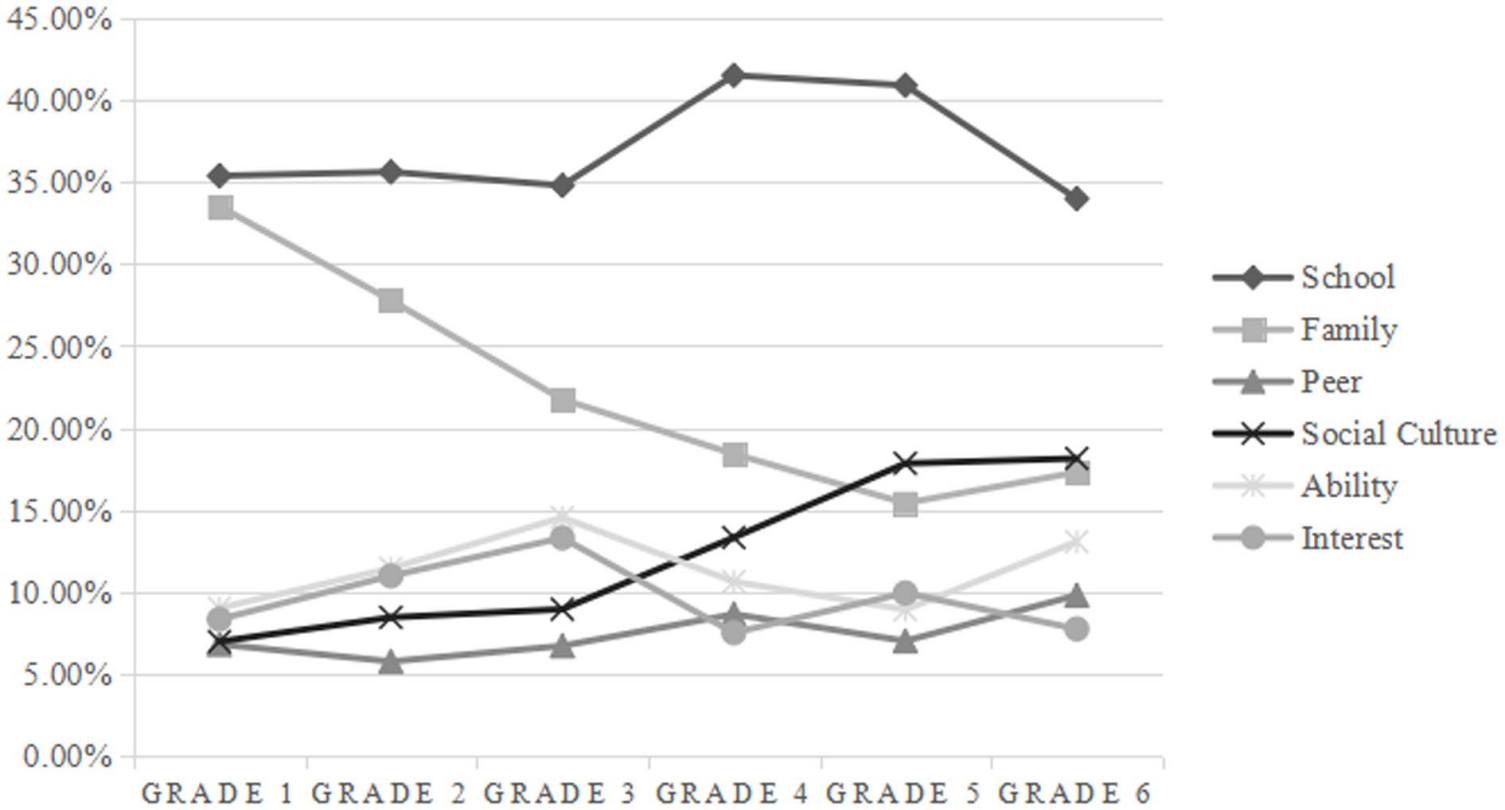
The percentage of topic texts with Grade Growth.

In terms of the percentage of texts on the topic, elementary school children mostly described two types of topics: school and family. The percentages of the six topics with increasing grade levels were shown in Figure 1. Specifically, the proportion of school topic remained high, family topic continued to decrease, social culture topic stayed up, and the proportion of peer, ability, and interest topics did not change much. In addition, grade 3 was an important time point for the distribution of topic proportions. After Grade 3, the proportion of school and social culture topics had increased significantly, and the proportion of ability and interest topics had changed from rising to falling. Comparing the proportion of each topic in Grade 1 and Grade 6, it was found that the grade 1 children were mainly concerned about the school and family topics, and in Grade 6, the difference in the proportion of each topic decreases.

### 4.2. Sentiment analysis results

Through sentiment analysis of the text data under different topics, the distribution of elementary school children’s sentiment tendency under different topics is shown in Table 3. The results showed that primary school children express more negative emotions on the topics of school (Negative: 48.7%), family (Negative: 42.7%), and social culture (Negative: 50.4%). At the same time, they expressed more positive emotions on the topics of the peer (Positive: 47.8%), ability (Positive: 46.5%), and interest (Positive: 48.4%).

**Table 3:** Results of topic sentiment distribution.

Perspective	Topic name	2 (Positive)	1 (Neutral)	0 (Negative)
Environmental system	School	611 (33.1%)	337 (18.2%)	899 (48.7%)
	Family	379 (33.6%)	267 (23.7%)	482 (42.7%)
	Peers	175 (47.8%)	107 (29.2%)	84 (23.0%)
	Social culture	190 (31.3%)	111 (18.3%)	306 (50.4%)
Individual factors	Ability	266 (46.5%)	143 (25.0%)	163 (28.5%)
	Interest	239 (48.4%)	118 (23.9%)	137 (27.7%)

The Kruskal-Wallis test was used to investigate differences in the sentiment expression of each topic across grades, as shown in Table 4. The results showed that primary school children have no significant sentiment expression difference on the three topics of school, family, and peer. In the topic of social culture, there was a significant difference in sentiment tendency between grade 6 (Positive: 22.7%; Negative: 62.0%) and grade 2 (Positive: 40.9%; Negative: 40.9%) and grade 3 (Positive: 41.7%; Negative: 37.5%). In the topic of ability, there was a significant difference in sentiment tendency between grade 1 (Positive: 30.2%; Negative: 45.3%) and grade 3 (Positive: 56.4%; Negative: 17.9%). In the topic of interest, there is a significant difference in sentiment tendency between grade 6 (Positive: 31.3%; Negative: 40.6%) and grade 2 (Positive: 58.7%; Negative: 24.5%). Combining the results of the sentiment tendencies for each of the three topics mentioned above across grade levels, we found that under the social culture topic, children had higher rates of negative emotions expressed in all the grades except for Grade 2 and 3. Under the ability topic, children expressed more positive emotions in all grades than Grade 1. Under the topic of interest, children showed more positive emotions in Grade 1–5, but more negative emotions in Grade 6.

**Table 4:** Changes in sentiment distribution of topics in different grades.

Topic name	Kruskal-Wallis test	Grade-grade comparison	Test statistics	Standard test statistics	Progressive significance
School	0.06				
Family	0.16				
Peers	0.66				
Social culture	0.00^**^	Grade 6-Grade 2	71.43	3.55	0.01
		Grade 6-Grade 3	79.68	3.47	0.01
Ability	0.01^**^	Grade 1-Grade 3	−95.45	−3.76	0.00
Interest	0.02^*^	Grade 6-Grade 2	69.66	3.52	0.01

^*^p < 0.05, ^**^p < 0.01.

In addition, the Kruskal-Wallis test was used to explore differences in sentiment expression between topics in each grade, as shown in Table 5. The results indicated significant differences in sentiment tendency for both school and peer topics in both junior and senior elementary grades, in which children showed relatively more negative emotions on school topics and relatively more positive emotions on peer topics. Also, in the junior elementary grades, children expressed more positive emotions on the interest topics than their emotional expressions on school and family topics. In addition, there was no significant difference in sentiment tendency of ability topic relative to the two topics of school and family in the junior elementary grades, but there were significant differences in the middle and senior elementary grades, in which children showed relatively more positive emotions on ability topic. Also, there were significant differences between the topics of social culture and interest in the junior grades. There were significant differences between the topics of social culture and peer and ability in the senior grades. Among them, children showed relatively more negative emotions on the social culture topic. Overall, there were differences in children’s sentiment tendencies on certain topics in different grades of primary school. In addition, compared with the middle grades of primary school, there were more differences in children’s emotional expression between the j junior and senior grades for different topics.

**Table 5:** The topic pairs with the significant pairwise comparison of sentiment in each grade.

Grade	Kruskal-Wallis test	Theme-theme	Test statistics	Standard test statistics	*p*
Grade 1	0.00^**^	School-peers	−81.27	−2.98	0.04^*^
		School-interest	−86.67	−3.46	0.01^**^
		Social culture-peers	105.99	3.02	0.04^*^
		Social culture-interest	−111.38	−3.33	0.01^**^
Grade 2	0.00^**^	School-peers	−165.18	−3.79	0.00^**^
		School-interest	−164.60	−4.92	0.00^**^
		Family-peers	−161.36	−3.64	0.00^**^
		Family-interest	−160.77	−4.65	0.00^**^
		Social culture-interest	−134.36	−3.03	0.04^*^
Grade 3	0.00^**^	School-ability	−131.62	−5.51	0.00^**^
		School-interest	−92.14	−3.73	0.00^**^
		Family-ability	−102.53	−3.95	0.00^**^
Grade 4	0.00^**^	Family-peers	−89.61	−3.04	0.04^*^
		Family-ability	−85.19	−3.09	0.03^*^
Grade 5	0.00^**^	School-peers	−136.12	−4.43	0.00^**^
		School-ability	−97.82	−3.52	0.01^**^
		School-interest	−94.56	−3.56	0.01^**^
		Social culture-peers	132.02	3.94	0.00^**^
		Social culture-ability	−93.71	−3.04	0.04^*^
		Social culture-interest	−90.46	−3.04	0.04^*^
Grade 6	0.00^**^	School-peers	−98.35	−3.53	0.01^**^
		School-ability	−102.21	−4.08	0.00^**^
		Family-peers	−100.85	−3.28	0.02^*^
		Family-ability	−104.71	−3.71	0.00^**^
		Social culture-peers	139.57	4.58	0.00^**^
		Social culture-ability	−143.43	−5.14	0.00^**^

^*^p < 0.05, ^**^p < 0.01.

## 5. Discussion

The purpose of this study is to analyze differences in the topic content of children’s concerns, variations in topics, and expression of sentiment tendencies in free-text writing at the elementary school to broaden the understanding of the cognitive and psychological developmental characteristics and needs of children at the elementary school. This paper used the LDA model to discuss the different topics of elementary school children. Then, it used sentiment analysis to compare the differences and variations of sentiment expression in different topics.

The results indicate that (1) Elementary free-text writing is divided into six topics: school, family, peer, and social culture in the environmental system, and ability and interest in the individual factor. (2) As the grade increases, the proportion of school topic remains high, the family topic continues to decline, social culture topic keeps rising, while the topics of peer, ability, and interest remain stable. (3) In the topics of school, family, social culture, and interest, elementary school children express relatively more negative emotions while in the topics of peer and ability they show relatively more positive emotions. And as the grade increases, the sentiment expressions negatively in the topic of social culture and positively in the topic of ability and interest. (4) There are more differences in children’s sentiment expressions on different topics in the junior and senior elementary grades.

### 5.1. Developmental trends in topics

Through the analysis of the LDA model, the results showed that the topics of primary school children in free-writing contained school, family, peer, social culture, ability, and interest. Children are most involved in two topics: school and family. The reason is that, in ecological systems theory, school and family are the two main microsystems of children (Bronfenbrenner, 1977). Primary school children spend a lot of time in school and family, so the focus of children’s attention is inevitably on school and family. School topics mainly include learning activities, teacher-student interaction, student–student interaction, and school activities. The family topics mainly involve family members and daily interactions.

Regarding the distribution of different topics in the six elementary grades, we found that as the grade increases, the proportion of school topic remains high, the proportion of family topic continues to decrease, the proportion of social culture topic keeps increasing, and the proportion of peer, ability and interest topics do not change much. The reason is that after entering primary school, children spend more time on course study and campus activities gradually instead of family life. Over time, children’s access to knowledge has also increased, and their attention has been gradually paid to social events and ethics. According to Kohlberg’s Moral Stages Theory (Gibbs, 1979), the sense of social morality is initially formed by preschool children and is further developed through school education in primary school. Generally speaking, junior-grade children experience society based on social responses, middle-grade children begin to be bound by strict social and moral rules, and senior-grades students have a life guide with abstract moral concepts. Primary school children in senior grades have gradually understood the meaning of the code of conduct under moral beliefs, being aware of their emotional performance and the possible adverse consequences for others or society, and they will restrain themselves under the requirements of morality and code of conduct. Therefore, the proportion of the social culture topic in the middle grade, that is, Grade 3, has shown a clear upward trend. A reasonable explanation for the decline in the proportion of the social culture theme in Grade 6 is that primary school children face the pressure of further education in this grade, so they have to devote time and energy to their studies. However, the proportion of peer topic is relatively small the possible reason is that it mainly involves children playing with their peers outside school, and interactions in school are included in the class, that is, the school topic.

### 5.2. Difference in sentiment expression

The results of the sentiment analysis indicate that elementary school children express emotions differently on different topics. In contrast, elementary school children express negative emotions more frequently on school, family, social culture, and interest topics, and positive emotions on peer and ability topics. Meanwhile, the results of the Kruskal-Wallis test indicate that as grade increases, shifts are found in elementary school children’s sentiment tendency on social culture, ability, and interest topics, mainly in the form of negative emotional expressions on social culture topics and positive emotional expressions on ability and interest topics.

Specifically, under the school topic, children express relatively more negative emotions overall. Potential explanations are as follows. In the early stage of primary school, children rely on teachers for care and guidance, but as they become more independent with growth, the weakening of the teacher-student relationship reduces children’s sense of belonging to school and their emotional connection with the society, leading to the increase of negative emotions. Two separate but related dimensions of conflict and closeness are often used to measure the quality of teacher-student relationships (TSRQ; Rudasill et al., 2010). Conflict represents the negativity that exists between teachers and students, while intimacy represents the level of support and warmth between teachers and students, as well as students’ willingness to approach and engage with teachers (Ladd and Burgess, 2001). The research showed that children, whether male or female, have less potential for intimacy with teachers and more conflicts over time in primary school (Spilt et al., 2012; Hajovsky et al., 2017). As a result, these conflicts can lead to emotional distress and limit children’s participation and performance in school and learning activities. Another possible explanation for this finding is that children’s social environment has become more complex and concerns about social assessments have increased, particularly concerning academic achievement. As grades increase, parents and teachers pay more and more attention to children’s academic performance, and the increase in academic stress can lead to an increase in children’s negative emotions. A self-reported study of children and adolescents aged 8–18 years showed that fear of social assessment and achievement assessment increases with age (Michiel Westenberg et al., 2004). Meanwhile, relative to Western children and adolescents, Chinese children, and adolescents report higher levels of fear of social evaluation, a phenomenon that is more common among children in the senior grades of primary school (Dong et al., 1994). The most common fears are often related to events such as failing exams and getting bad grades. Interestingly, children show more positive emotions overall when describing their homework on the topic of school. Given that homework is associated with after-school time, text that involves free breaks and family companionship can promote positive emotions.

Under the family topic, children show relatively more negative emotions overall. The ecological systems theory supports the family as an important microsystem affecting children’s social and emotional development. Parent–child relationship quality is one of the key environmental determinants or correlates of children’s well-being (Brumariu, 2015). Parent–child relationships tend to undergo major changes during childhood and adolescence. Specifically, parents and children experience more intense conflicts, express less physical affection, spend less time together, and have less intimacy with each other (Hazel et al., 2014). The topic modeling results indicate that the proportion of texts with family topics decreases with grade level. This phenomenon may be a manifestation of children spending less time with their parents and their emotional distance increasing, so emotional expressions become less positive. This finding is consistent with Larson and Richards (1991), which found a decrease in family time spent together from late childhood to early adolescence, which was associated with an increase in family reports of negative emotions. Another possible explanation for this finding is that parental emphasis and pressure on high levels of academic achievement leads to higher levels of conflict and negativity. In China, there is a perception that academic achievement is closely linked to future achievement, and shame is used as a disciplinary strategy in parenting styles (Leung et al., 1994). When children’s academic achievement falls short of parents’ expectations, parents’ strategies lead to increased negativity.

Under the peer topic, children show relatively more positive emotions overall. After entering primary school, children become increasingly independent from the development pattern of receiving care from adults and turn to reliance on peers for social support and companionship (Leung et al., 1994). Building friendships with peers is a significant development task during childhood and adolescence (Poulin and Chan, 2010). Good peer relationships can give children more emotional support, emotional responsiveness, and a sense of well-being, while individuals with friendships of junior quality levels are more likely to experience negative emotions such as loneliness and anxiety (Valiente et al., 2020). Under the peer topic, playing with peers during recess and outside school can promote an increase in children’s positive emotions. This finding is consistent with (Larson and Richards, 1991) that children and adolescents with peers, especially outside the home or school experience higher positive emotions. In addition to the interaction with peers, it also contains many natural experiences, such as “spring,” “forest,” and “birds.” Research has shown that nature experiences can help children reduce anxiety, rumination, and negative emotions while retaining positive emotions (Bratman et al., 2015; Mygind et al., 2019).

Under the social culture topic, children express relatively more negative emotions overall and express relatively more negative emotions in the senior grades. Most text on this topic describes social news dominated by negative events, such as littering, environmental pollution, natural disasters, and terrorist attacks, and children describe the events with details and express their attitudes. A study of children aged 8–13 years showed that most children were aware of the reality of television news and produced fear, frustration, and other negative reactions (Kandemir-Ozdinc and Erdur-Baker, 2013). This suggests that although children do not have enough critical thinking about news reporting compared with adolescents and adults, children can recognize the seriousness of negative news and generate negative emotions. Negative bias means that individuals process stimuli of different emotional valence asymmetrically, and compared with positive or neutral stimuli, negative stimuli were proved to obtain more children’s attention and cognitive processing (Vaish et al., 2008). There is ample empirical evidence that young children were more sensitive to negative emotional information and recall negative emotional stories better than positive and neutral emotional stories in delayed recall (Baltazar et al., 2012; Van Bergen et al., 2015). Therefore, children’s memory of negative social behaviors during free writing recall higher negative emotions than positive or neutral social behaviors, which may also lead to more negative affective expressions in senior grades.

Under the ability and interest topic, children express more positive emotions overall. Interest is one of the triggers for positive emotions. The study finds that on this topic, most children describe their liking and positive emotions about the natural environment, toys, and food. This indicates that children actively explore things in the external environment and the process produces g good psychological states. According to the four-phase model of interest development (Hidi and Renninger, 2006), young children’s interest is likely to be characterized primarily by situational interest. After experiencing a variety of situational interests, their long-term personal interest begins to emerge. As personal interests are fully developed, the connections between tasks, emotions, values, goals, and beliefs become stronger. Under the ability topic, we found positive expressions of children’s emotions about academic-related skills, talents (e.g., piano, singing), and sports (e.g., running, basketball). Early elementary school children’s motivation to engage in activities primarily involves interest and utility/importance, and middle and late elementary school children value the achievement value/personal importance, interest, and utility value of the activity (Wigfield and Cambria, 2010). When they value an activity, they can engage in it and stick to it for a long time. At the same time, individuals expect to do well in activity and value it, and they experience emotions such as hope and joy. If not, they will suffer anxiety and despair (Pekrun, 2006). Thus, children show relatively positive emotions overall under the ability and interest topics. Under the topic of interest, children show relatively more negative emotions in grade 6 except for a food waste phenomenon in the class in the food topic, which was discussed and criticized by the class teacher, and most of the students describe the causes and consequences of the incident and their reflections.

Interestingly, by using the Kruskal-Wallis test to analyze differences in the expression of sentiment tendencies between topics within the same grade, we found more differences in children’s expression of emotions between topics in the junior and senior grades relative to the middle grades of elementary school. According to Piaget’s cognitive development theory, children in the preoperational stage are likely to express more extreme emotions because their cognition, vocabulary, and writing skills limit their emotional expression to a certain extent. By the time they enter the concrete operational stage, children are no longer simply concerned with appearances and can think logically about objective things and experiences. Meanwhile, as cognitive enrichment and experience increase, children’s language skills and emotional understanding improve significantly and they can use more abstract, emotionally charged terms to express their emotions accurately (Pons and Harris, 2005). This may amplify the diversity and variability of connections between different topic situations and emotions, leading to increasingly significant differences between emotions across topics. Furthermore, consistent with Larson et al. (2002) in which senior elementary school children were exposed to increased academic stress, post-school environmental changes, and interpersonal changes, they would have junior emotional range and express more extreme emotions in adolescence.

## 6. Contributions and limitations

The contributions of this paper are as follows. First, compared to the possible contextual limitations and problem bias of data in common studies, this paper uses free-writing texts spanning grade 1 to grade 6 as a data source, and uses text mining for topic classification and sentiment analysis, providing a new data source and enriching research methods for studies related to children’s psychological development. Second, this paper discovers children’s attentional characteristics in daily life based on topic modeling, and longitudinally studies the changing trends of topics among elementary school children from grade 1 to grade 6, which helps to enrich the study of cognitive development patterns and characteristics of elementary school children. At last, the empirical results on sentiment tendencies and developmental differences under different topics could supposedly reveal children’s emotional developmental needs for different topics in elementary schools and enrich the research field of sentiment analysis.

Our research results have a great practical significance, hopefully, to promote the construction of primary school mental health counseling. First, we use the LDA model to identify free-writing topics that are useful for parents and teachers to guide children in different grades. Parents and teachers can explain and suggest topics that are interesting or understandable to children to effectively guide children to recognize and understand life experiences. Also, it helps parents and teachers to keep abreast of children’s interests and improve the quality of nurturing. Second, it helps to effectively meet children’s emotional development needs through the sentiment analysis of different topics. When children’s emotional state fluctuates significantly, parents and teachers can understand the potential influences that may exist and guide them in time to avoid deterioration. In their daily interactions, parents and teachers are better able to interact with children and help them maintain positive attitudes in a more effective and personalized manner. Finally, this study offers potential strategies for negative emotion management. Families and schools should pay more attention to the emotional state of their senior-grade students. By providing adequate support and relief for academic stress in the senior grades, a positive competitive climate can be developed to mitigate the growth of negative emotions. These strategies will help improve parent and teacher-student interactions and contribute to the psychologically healthy development of elementary school children.

Due to the limitations of the conditions, this paper only obtained free-writing text data from 18 students in the same class. In future research, we will consider expanding the scope and number of research subjects and analyzing the relevant contents in the context of the current social background to further test the research methods and results. At the same time, in the future, the analysis of gender differences can be considered to study the attention differences and change trends of children of different genders on different topics, as well as the differences in emotional expression, to realize the dynamic expansion of this research.

## Data availability statement

The original contributions presented in the study are included in the article/supplementary material, further inquiries can be directed to the corresponding authors.

## Ethics statement

The studies involving human participants were reviewed and approved by Hubei University, School of Education, Science Ethics Committee. Written informed consent to participate in this study was provided by the participants’ legal guardian/next of kin.

## Author contributions

ML, XJ, and BZ contributed to the study design, theoretical basis, data analysis, and discussion of the manuscript. GY contributed to the acquisition, collection and sorting of data. GL, NJ, DW, and ZZ contributed to the discussion and conclusions of the manuscript and critically revised the article. TS contributed to the revision and touch-up of the manuscript. All authors contributed to the article and approved the submitted version.

## Funding

This work was supported by the National Natural Science Foundation of China “Research on the Security Management Mechanism of Comprehensive Quality Evaluation Data for the New College Entrance Examination: Blockchain Technology Empowerment Perspective” (Grant number: 72204077), the General Project of the Natural Science Foundation of Hubei Province “Based on Blockchain New College Entrance Examination Comprehensive Quality Evaluation Data Security Management Mechanism” (Grant number: 2021CFB470), Hubei University Teaching Reform Research Project “Research on Blockchain-Based Normal Student Course Archive Data Security Management Mechanism” (Grant number: 090017168), and Hubei University Teaching Reform Research Project Supported by the reform research project “Research on Data Security Management Mechanism of Postgraduate Training Process Empowered by Blockchain” (Grant number: 090014534).

## Conflict of interest

ZZ was employed by China Unicom Hubei Branch.

The remaining authors declare that the research was conducted in the absence of any commercial or financial relationships that could be construed as a potential conflict of interest.

## Publisher’s note

All claims expressed in this article are solely those of the authors and do not necessarily represent those of their affiliated organizations, or those of the publisher, the editors and the reviewers. Any product that may be evaluated in this article, or claim that may be made by its manufacturer, is not guaranteed or endorsed by the publisher.

## Footnotes

^1^ https://www.paralleldots.com/emotion-analysis
